# Cadmium exposure and risk of pancreatic cancer: Systematic review and meta-analysis

**DOI:** 10.1371/journal.pone.0319283

**Published:** 2025-04-29

**Authors:** Yaser Soleimani, Mohammad Nayebi, Sheyda Mahmoudi, Mahdi Daraei, Soroush Khorsand, Mohammad amin Jahazi, Maryam Yadollahi Farsi, Fatemeh Khalafi, Mahdieh Varseh, Zahra Mousavi Jarrahi, Mahna Soleimani, Kamelia Massoudnia, Saeideh Karamian, Alireza Mosavi Jarrahi

**Affiliations:** 1 Medical School, Shahid Beheshti University of Medical Sciences, Tehran, Iran; 2 Student Research Committee, Khomein University of Medical Sciences, Khomein, Iran; 3 Department of Medical Laboratory Sciences, Shahroud University of Medical Sciences, Shahroud, Iran; 4 Cancer Research Centre, Shahid Beheshti University of Medical Sciences, Tehran, Iran; University of Southern California, UNITED STATES OF AMERICA

## Abstract

**Background:**

Pancreatic cancer is one of the most lethal malignancies, characterized by a poor prognosis and limited treatment options. Increasing evidence suggests that environmental factors, including heavy metal exposure, play a role in pancreatic cancer development. Cadmium, a toxic heavy metal classified as a Group 1 carcinogen, has been implicated in cancer progression. However, its association with pancreatic cancer remains uncertain, with conflicting results from epidemiological studies. This systematic review and meta-analysis aim to assess the association between cadmium exposure and the risk of pancreatic cancer.

**Methods:**

A comprehensive literature search was conducted using multiple databases to identify studies that explored the relationship between cadmium exposure and pancreatic cancer risk. Eleven eligible studies were included in the meta-analysis. Pooled odds ratios (OR) with 95% confidence intervals (CI) were calculated to estimate the overall effect size. Heterogeneity was assessed using I², T², and Cochran’s Q-test. Publication bias was evaluated using Egger’s and Begg’s tests.

**Results:**

The pooled analysis revealed a significant association between cadmium exposure and the risk of pancreatic cancer, with an overall OR of 2.01 (95% CI: 1.30, 2.72), indicating that individuals exposed to cadmium had more than twice the risk of developing pancreatic cancer compared to those with lower or no exposure. Substantial heterogeneity was observed across studies (I² = 98.08%, T² = 1.37, H² = 52.21). Cochran’s Q-test also indicated significant heterogeneity (Q(10) = 304.52, p = 0.00). Egger’s test (p = 0.5040) and Begg’s test (p = 1.0000) showed no evidence of publication bias.

**Conclusion:**

This meta-analysis demonstrates a significant positive association between cadmium exposure and an increased risk of pancreatic cancer. Despite the considerable heterogeneity across studies, the findings suggest that cadmium is a potential environmental risk factor for pancreatic cancer. Further research is required to explore the underlying biological mechanisms and to develop strategies for reducing cadmium exposure, particularly in high-risk populations.

## Introduction

Pancreatic cancer is one of the most aggressive and lethal malignancies, with a poor prognosis and limited treatment options. It is the seventh leading cause of cancer-related deaths worldwide [1], with a five-year survival rate of less than 10%, primarily due to late-stage diagnosis and resistance to standard therapies. While genetic predisposition and lifestyle factors such as smoking, alcohol consumption, and obesity are recognized contributors, the role of environmental exposures in pancreatic cancer etiology has garnered increasing attention in recent years. One environmental factor that has come under scrutiny is exposure to heavy metals, particularly cadmium, due to its known carcinogenic properties [2].

Cadmium is a naturally occurring heavy metal found in the earth’s crust, but its levels in the environment have significantly increased due to anthropogenic activities such as mining, smelting, battery manufacturing, and the use of phosphate fertilizers [3]. Human exposure to cadmium primarily occurs through contaminated food, tobacco smoke, and occupational hazards in industrial settings. Once absorbed, cadmium accumulates in various organs, including the liver, kidneys, and pancreas [4–6], and has a biological half-life of more than 10 years [7], making chronic exposure a significant public health concern. Importantly, the International Agency for Research on Cancer (IARC) has classified cadmium as a Group 1 carcinogen, indicating sufficient evidence of its carcinogenicity in humans.

Mechanistically, cadmium is thought to contribute to carcinogenesis through several pathways, including the generation of reactive oxygen species (ROS), induction of chronic inflammation [8–10], interference with DNA repair mechanisms, and disruption of normal cell signaling pathways. These effects can lead to DNA damage, mutations, and uncontrolled cell proliferation, all of which are hallmarks of cancer development. Although the link between cadmium exposure and certain cancers—such as lung, kidney, and prostate cancer—has been well established [11], its role in pancreatic cancer remains less clear. Epidemiological studies investigating the relationship between cadmium and pancreatic cancer have yielded conflicting results, with some studies suggesting a positive association and others finding no significant link.

The pancreas is particularly susceptible to oxidative stress and inflammatory damage [12,13], making it a plausible target for cadmium-induced carcinogenesis. However, the variability in study designs, cadmium exposure measurements, and confounding factors in previous research has led to inconclusive findings. Additionally, the biological mechanisms through which cadmium may influence pancreatic tumorigenesis have not been fully elucidated [14], further complicating our understanding of this potential risk factor.

Given the significant burden of pancreatic cancer and the widespread exposure to cadmium, it is critical to systematically evaluate the available evidence to determine whether there is a definitive association between cadmium exposure and pancreatic cancer risk. A systematic review and meta-analysis provide an ideal approach to synthesizing existing data, allowing for the pooling of results from multiple studies to increase statistical power and assess the consistency of findings across different populations and settings. This method also enables the identification of potential sources of heterogeneity and the evaluation of dose-response relationships, which are essential for understanding the magnitude and direction of the association.

In this systematic review and meta-analysis, we aim to comprehensively assess the relationship between cadmium exposure and the risk of pancreatic cancer by analyzing data from epidemiological studies. By doing so, we seek to clarify the potential role of cadmium as a risk factor for pancreatic cancer, provide insights into the biological plausibility of this association, and highlight gaps in the current literature that warrant further investigation. Ultimately, the findings of this study may inform public health policies and strategies aimed at reducing cadmium exposure and mitigating its impact on pancreatic cancer incidence.

## Materials and methods

### Study design and selection criteria

A comprehensive detail of the protocol of this study has been already published [15]. This systematic review and meta-analysis was conducted in accordance with the Preferred Reporting Items for Systematic Reviews and Meta-Analyses (PRISMA) guidelines to ensure a comprehensive and transparent review process. The objective was to assess the association between cadmium exposure and pancreatic cancer risk, focusing on human observational studies. Eligible studies included case-control and cohort designs, as these provide the most robust data for investigating potential causal relationships between exposure and disease outcomes.

Inclusion criteria were:

1. Studies that assessed cadmium exposure, either through environmental or occupational sources, and reported on the incidence of pancreatic cancer.
2. Studies that provided sufficient data to calculate risk estimates, such as odds ratios (OR), or hazard ratios (HR), with 95% confidence intervals (CI).
3. Peer-reviewed articles published in any language, with a focus on human populations.

Exclusion criteria included:

1. Studies that lacked adequate data on cadmium exposure.
2. Reviews, editorials, case reports, or animal studies.
3. Studies where cadmium was measured indirectly or where other confounding heavy metals were not adequately controlled.

### Search strategy

The search strategy for this study involved conducting a comprehensive literature search across multiple electronic databases, including PubMed, Scopus, Web of Science (WOS), and Embase, from their inception until May 1, 2024. The search strategy was formulated with the assistance of an expert librarian and involved a combination of Medical Subject Headings (MeSH) and free-text terms. Specific search terms used included variations of “Cadmium” and “Cancer” or “pancreatic cancer.” The search yielded 2,898 articles from PubMed, using the search terms (Cadmium) AND (“Cancer” OR “pancreatic cancer”). Scopus returned 6,089 articles with the query TITLE-ABS-KEY (“Cadmium”) AND TITLE-ABS-KEY (“Cancer” OR “pancreatic cancer”). In Web of Science, 3,701 articles were identified with the terms (TS=(“Pancreatic cancer” OR “Cancer”)) AND TS=(Cadmium). To ensure the inclusion of all relevant studies, the search was further supplemented by manually screening the reference lists of all selected studies and reviews. Additionally, conference abstracts and grey literature were reviewed, where available, to capture any potentially overlooked research.

### Information selection and extraction

Titles and abstracts retrieved from the database search were independently screened by two reviewers to assess their eligibility based on predefined inclusion and exclusion criteria. In cases of disagreement, the reviewers reached a consensus or consulted a third independent reviewer to resolve any conflicts. Full texts of studies deemed potentially relevant were obtained for further evaluation. Data extraction was conducted using a standardized form, capturing key variables such as study design (e.g., case-control, cohort), sample size, geographic location, and population characteristics, including age, gender, and the nature of exposure (occupational vs. environmental). Additionally, methods used to assess cadmium exposure (e.g., urine cadmium levels or occupational records) were recorded. Risk estimates, such as odds ratios (OR), relative risks (RR), or hazard ratios (HR) for pancreatic cancer, along with corresponding 95% confidence intervals (CIs), were also extracted. Furthermore, adjustments for potential confounding variables, such as smoking, age, and alcohol consumption, were noted. When multiple risk estimates were available, those adjusted for the greatest number of confounders were selected for meta-analysis. Any missing or unclear data were clarified by contacting the original study authors.

### Scientific evidence

The methodological quality of the included studies was assessed using the Joanna Briggs Institute (JBI) Critical Appraisal Tools, which are specifically designed to evaluate the risk of bias in observational studies. The JBI tools assess various domains of study quality, including the adequacy of the study’s exposure assessment, appropriateness of the control group, consideration of confounding factors, and validity of outcome measures.

Each study was scored based on these criteria, and only those with moderate to high-quality scores were included in the meta-analysis. Studies with high risk of bias were excluded from the quantitative synthesis but were qualitatively discussed. To ensure reliability, quality appraisal was performed independently by two reviewers, with discrepancies resolved through discussion.

### Meta-analysis

Meta-analysis was performed using STATA version 17 (StataCorp, College Station, TX), applying a random-effects model to account for between-study heterogeneity. The choice of a random-effects model was made based on the assumption that the studies were not functionally identical due to differences in populations, methods of cadmium exposure assessment, and study designs.

The primary outcome was the pooled estimate of the association between cadmium exposure and pancreatic cancer risk, expressed as a summary OR or HR with 95% CIs. Heterogeneity across studies was assessed using the I² statistic, with values greater than 50% indicating substantial heterogeneity. Cochran’s Q test was also used to assess the significance of heterogeneity (p < 0.10).

Subgroup analyses were performed to explore potential sources of heterogeneity. This subgroup analysis is performed according to the year of publication.

Sensitivity analyses were conducted by excluding low-quality studies and those that contributed significantly to heterogeneity, to assess the robustness of the pooled estimates.

### Stata application (Version 17)

All statistical analyses were performed using STATA version 17. The “metan” command was used to pool risk estimates across studies and generate forest plots. The degree of publication bias was examined using funnel plots, and Egger’s regression test was applied to assess the asymmetry of the funnel plot. A p-value of less than 0.05 was considered indicative of significant publication bias.

Additionally, the “metafunnel” command was utilized to visualize publication bias, and the “metabias” command provided statistical tests for small-study effects. Meta-regression analyses were conducted to explore the impact of covariates, such as study quality and geographic region, on the overall risk estimate.

## Results

**Fig 1:**
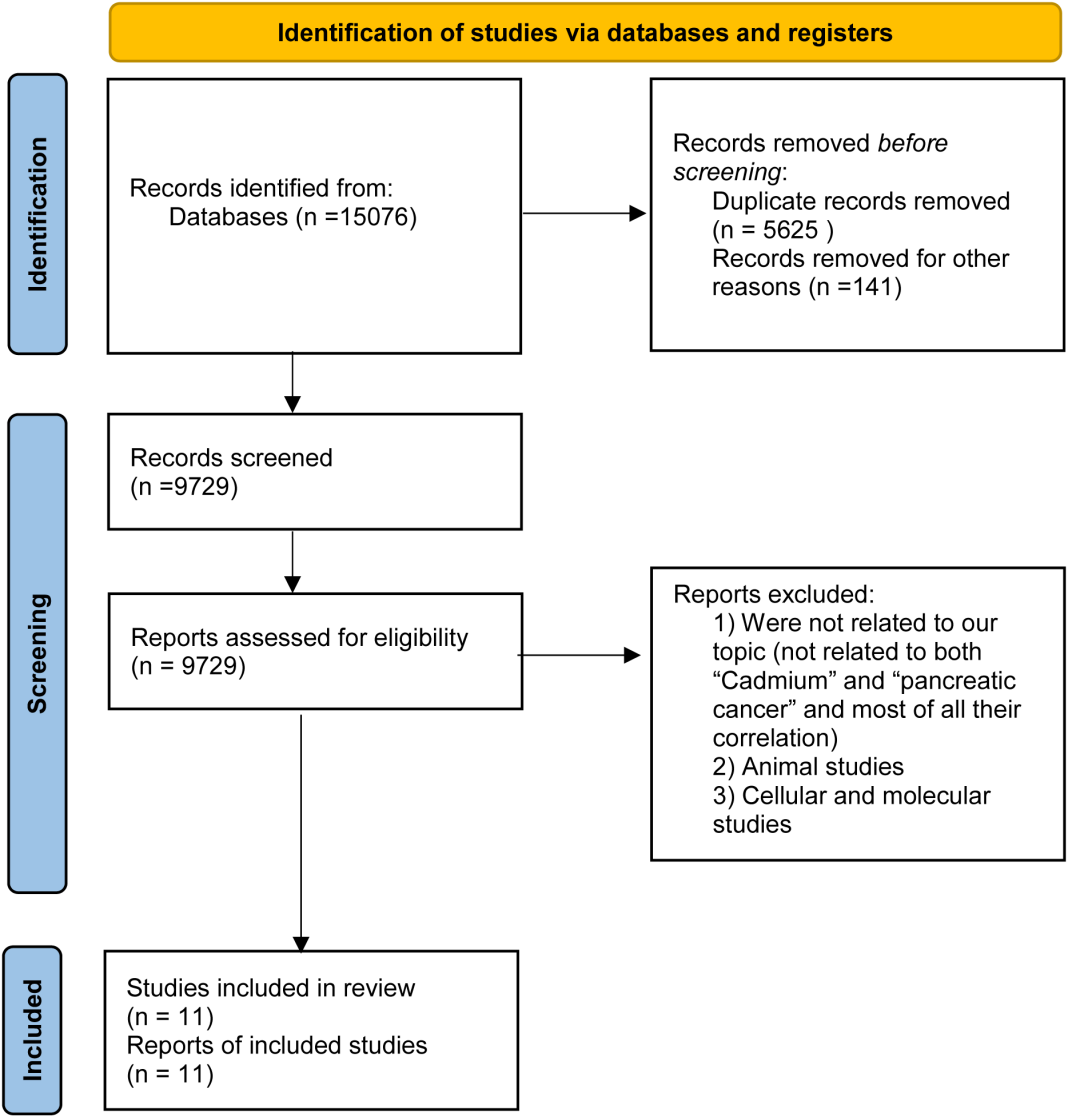
Flow diagram of systematic review and meta-analysis, cadmium exposure and risk of pancreatic cancer.

**Fig 2:**
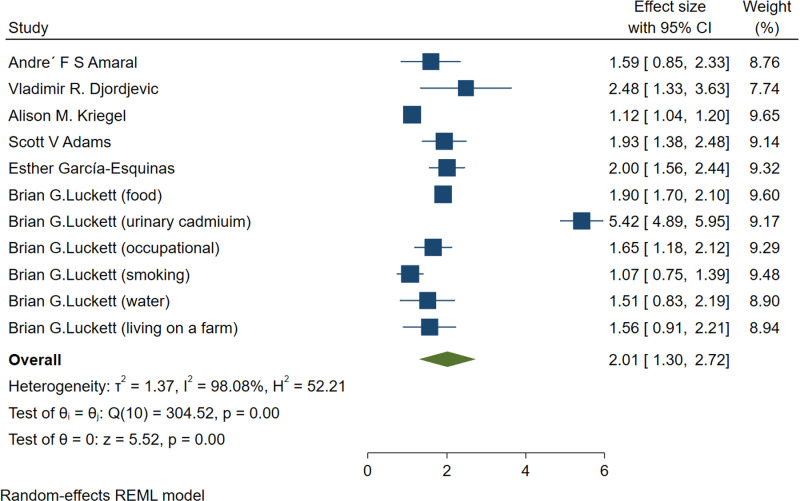
Forest plot, meta-analysis of cadmium exposure and risk of pancreatic cancer.

This meta-analysis included 11 studies ( and ) out of 15,076 articles that investigated the relationship between cadmium exposure and the risk of pancreatic cancer. Reasons for study exclusion are detailed in the Preferred Reporting Items for Systematic Reviews and Meta-Analyses (PRISMA) flow diagram (Fig 1). The study which is written by Brian G Luckett [16], has been divided ORs and 95% CIs due to the subtypes which the way of exposures were different. The pooled results indicate a significant positive association between cadmium exposure and pancreatic cancer risk. The overall odds ratio (OR) was 2.01, with a 95% confidence interval (CI) ranging from 1.30 to 2.72 (Fig 2). This suggests that individuals with higher cadmium exposure are more than twice as likely to develop pancreatic cancer compared to those with lower or no exposure. The lower bound of the confidence interval (1.30) is greater than 1.0, indicating a statistically significant association, with a p-value less than 0.05.

**Table 1:** Summary of published results on the relationship between exposure to cadmium and risk of pancreatic cancer.

Reference	Quality Score	Country	Study Design	Source of funding	Age range	Exposure Assessment Measurement Method	Diagnostic Criteria Used	Follow up Duration	Reported Results(Odds Ratio and95% Confidence Intervals)
Brian G.Luckett (food) [16]	Low Risk	USA	case-control	Louisiana State Board of Regents	>20 Years	questionnaire- laboratory examination	laboratory examination(urine sample)	2001–2005	OR: 1.90 95% CI: (1.88–2.82)
Brian G.Luckett (urinary cadmium) [16]	Low Risk	USA	case-control	Louisiana State Board of Regents	>20 Years	questionnaire- laboratory examination	laboratory examination(urine sample)	2001–2005	OR: 5.42 95% CI: (3.02–8.81)
Brian G.Luckett (occupational) [16]	Low Risk	USA	case-control	Louisiana State Board of Regents	>20 Years	questionnaire- laboratory examination	laboratory examination(urine sample)	2001–2005	OR: 1.65 95% CI: (1.03–1.29)
Brian G.Luckett (smoking) [16]	Low Risk	USA	case-control	Louisiana State Board of Regents	>20 Years	questionnaire- laboratory examination	laboratory examination(urine sample)	2001–2005	OR: 1.07 95% CI: (0.75–1.42)
Brian G.Luckett (water) [16]	Low Risk	USA	case-control	Louisiana State Board of Regents	>20 Years	questionnaire-laboratory examination	laboratory examination(urine sample)	2001–2005	OR: 1.51 95% CI: (0.77–2.97)
Brian G.Luckett (living on a farm) [16]	Low Risk	USA	case-control	Louisiana State Board of Regents	>20 Years	questionnaire- laboratory examination	laboratory examination(urine sample)	2001–2005	OR: 1.56 95% CI: (0.81–3.01)
Andre´ F S Amaral [17]	Low Risk	Spain	Case-Control	^*^	Not mentioned	Interview-questionnaire, clinical history	Hospital	1992-2005	OR:1.59 95% CI: (0.88–3.87)
Vladimir R. Djordjevic [18]	Low Risk	Serbia	case-control	The project was partly supported by the Ministry of Education, Science and Technological Development of Serbia and the Oklahoma State University Center for Health Science Pilot Grant Program	All	Laboratory examination	Human observational, experimental and in vitro studies	2014–2016	OR: 2.48 95% CI: (0.93–9.22)
Alison M. Kriegel [19]	Low Risk	Egypt	case-control	^*^2	All	Laboratory examination and Histopathologic examination, Interview-questionnaire	laboratory examination(serum cadmium)	2001–2002	OR: 1.12 95% CI: (1.04–1.23)
Esther García-Esquinas [20]	Low Risk	USA	Cohort	^*^3	45-75 years	questionnaire and physical examination	each state’s Department of Health	1990–2008	OR: 2.00 95% CI: (1.29–3.11)
Scott V Adams [21]	Low Risk	USA	Cohort	SVA was supported in part by NIH National Cancer Institute Cancer Prevention Training Grant, and an ASPO/ASCO Cancer Prevention Fellowship sponsored by the Prevent Cancer Foundation	≥17 years	interviews and examinations	Not mentioned	Not mentioned	OR: 1.93 95% CI: (1.50–4.53)

*This work was partly supported by the Association for International Cancer Research (AICR09–0780), Fondo de Investigacio ´n Sanitaria, Spain (#PI09–02102), Red Tema´tica de Investigacio ´n Cooperativa en Ca´ncer (RTICC) and CIBER de Epidemiologı´a y Salud Pu´blica (CIBERESP), Instituto de Salud Carlos III, Ministry of Health, Spain, Fundacio ´n Cientı´fica de la Asociacio ´n Espan~ola Contra el Ca´ncer (AECC) and the Intramural Research Program of the Division of Cancer Epidemiology and Genetics, National Cancer Institute, USA. The Dartmouth Trace Element Core is partly supported by NIH grant number P42 ES007373 from the National Institute of Environmental Health Sciences.

*2 This work was supported by Eli Lilly Research; the Topfer Research fund from the M.D. Anderson Cancer Center; National Cancer Institute grants CA K07 090241 and R03 CA099513-01; and University of Michigan Cancer Center Support grant 5 P30 CA46592 to A.S.S. Stipend support for A.M.K. was provided by the Office of Science (Biological and Environmental Research), U.S. Department of Energy (grant DE-FG02-98ER62704 to D.A.B.).

*3 This work was supported by grants from the National Heart Lung and Blood Institute (HL090863) and the Strong Heart Study (HL41642, HL41652, HL41654, and HL65521). E.G.-E. was supported by a Río Hortega (CM10/00332) research training grant from the Spanish Ministry of Economy and Competitiveness (Carlos III Institute of Health) and by the Enrique Nájera predoctoral grant awarded by the Spanish Society of Epidemiology and funded by the National School of Public Health. J.G.U. is employed by MedStar Health Research Institute, Hyattsville, Maryland. L.G.B. is employed by Missouri Breaks Industries Research Inc., Timber Lake, South Dakota.

**Table 2:** Patients with leukemia according to their occupational exposure from studies included in the meta-analysis.

Reference	Number of cases	Number of controls	Sample size (No. of cases + No. of controls)
Andre´ F S Amaral [17]	114	398	512
Vladimir R. Djordjevic [18]	31	29	60
Alison M. Kriegel [19]	31	52	83
Esther García-Esquinas [20]	–	–	3792
Scott V Adams [21]	–	–	20,024
Brian G. Luckett (food) [16]	69	158	227
Brian G. Luckett (urinary cadmium) [16]	69	158	227
Brian G. Luckett (occupational) [16]	69	158	227
Brian G. Luckett (smoking) [16]	69	158	227
Brian G. Luckett (water) [16]	69	158	227
Brian G. Luckett (living on a farm) [16]	69	158	227

### Subgroup analysis

A subgroup analysis was performed to examine whether the association between cadmium exposure and pancreatic cancer risk varied according to the year of publication. The effect size for the studies included in this analysis was reported as odds ratio (OR), with the standard error (SE) provided for each estimate. The heterogeneity of the studies was assessed using several statistical metrics, including Tau-squared (T²), I-squared (I²), and H-squared (H²). The heterogeneity was substantial, with a T² value of 1.37, an I² value of 98.08%, and an H² value of 52.21, indicating considerable variability among the studies. A test of homogeneity between studies (θi = θj) showed significant heterogeneity (Q(10) = 304.52, p = 0.00), further supporting the need for subgroup analysis to explore potential sources of this variability. The test of group differences (Qb(4) = 30.24, p = 0.00) demonstrated significant variation in the effect sizes between studies based on their year of publication. This indicates that the year of publication may be a moderating factor influencing the reported association between cadmium exposure and pancreatic cancer risk.

explanations for this observed variability could include changes in study design, exposure assessment methods, or advancements in diagnostic techniques over time.

In conclusion, the subgroup analysis according to publication year suggests that the relationship between cadmium exposure and pancreatic cancer may be influenced by temporal factors, warranting further investigation into how methodological advancements over time might impact reported associations.

### Heterogeneity

**Fig 3:**
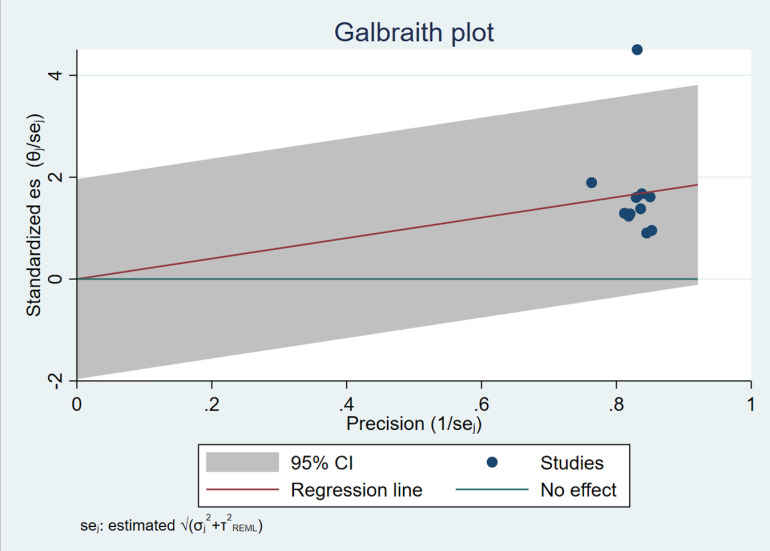
Galbraith plot, demonstrating heterogeneity.

**Fig 4:**
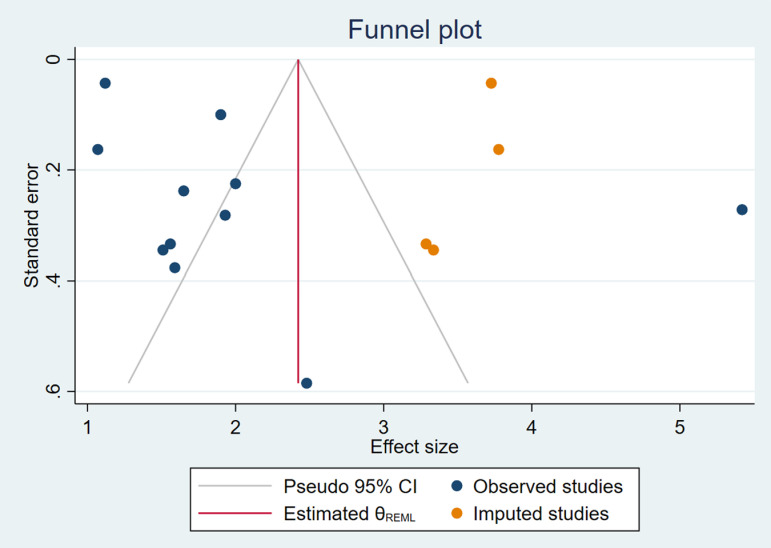
Funnel plot, observed and imputed studies.

A key finding of this analysis is the substantial heterogeneity among the included studies. The heterogeneity measures were as follows: T² = 1.37, I² = 98.08%, and H² = 52.21 (Figs 3 and 4). The high I² value of 98.08% suggests that nearly all the variation in effect sizes across studies can be attributed to heterogeneity rather than chance. This degree of heterogeneity implies significant differences in study populations, methodologies, exposure assessment methods, or other underlying factors across the included studies. Such variation raises concerns about the generalizability of the pooled estimate and suggests the need for further investigation into potential sources of heterogeneity. The Cochran’s Q-test for heterogeneity further supports this observation, with Q(10) = 304.52 and a p-value of 0.00. The significant p-value indicates that the differences in effect sizes across studies are unlikely to be due to random variation alone. This emphasizes the importance of accounting for differences in study design and population characteristics when interpreting the overall findings.

### Test of overall effect

The test of the overall effect, which evaluates whether the pooled effect size differs significantly from zero (θ = 0), revealed a z-value of 5.52 and a p-value of 0.00. This highly significant result confirms the strong positive association between cadmium exposure and pancreatic cancer risk. In practical terms, this finding means that, across the included studies, individuals exposed to cadmium were consistently found to have an increased likelihood of developing pancreatic cancer compared to unexposed individuals. This strengthens the evidence that cadmium may be a risk factor for pancreatic cancer.

### Publication bias

To evaluate potential publication bias, both Egger’s and Begg’s tests were conducted to assess whether smaller studies with potentially non-significant results were less likely to be published, which could skew the results of the meta-analysis.

The Egger test for small-study effects yielded a beta1 coefficient of 1.83 with a standard error (SE) of 2.735, a z-value of 0.67, and a p-value of 0.5040. Since the p-value is greater than 0.05, there is no statistically significant evidence of small-study effects. This suggests that smaller studies were not disproportionately contributing to the observed association, and the findings of the meta-analysis are unlikely to be biased by the selective publication of positive results.

Similarly, the Begg’s test for small-study effects, which uses Kendall’s score, indicated no evidence of publication bias. The test yielded a Kendall’s score of 1.00, with a standard error of 12.845, a z-value of 0.00, and a p-value of 1.0000. This result further corroborates the findings from the Egger test, reinforcing the conclusion that publication bias is unlikely to have significantly influenced the results of this meta-analysis.

### Summary of findings

In summary, the results of this systematic review and meta-analysis provide strong evidence of a significant association between cadmium exposure and an increased risk of pancreatic cancer, with a pooled OR of 2.01 (95% CI: 1.30, 2.72). While the association is robust, the high degree of heterogeneity among the included studies highlights the need for caution in interpreting the findings. The heterogeneity likely reflects differences in study design, population characteristics, cadmium exposure assessment methods, and potential confounders. Despite this, the consistency of the positive association across the studies suggests that cadmium may be an important environmental risk factor for pancreatic cancer.

Moreover, the lack of evidence for small-study effects or publication bias, as indicated by both the Egger and Begg tests, provides further confidence in the validity of the findings. These results underscore the need for further research to better understand the mechanisms by which cadmium may contribute to pancreatic carcinogenesis and to explore ways to mitigate exposure, particularly in populations at high risk.

## Discussion

The findings of this systematic review and meta-analysis provide strong evidence of a significant association between cadmium exposure and an increased risk of pancreatic cancer, with a pooled odds ratio of 2.01, indicating that individuals exposed to cadmium are more than twice as likely to develop pancreatic cancer compared to those with lower or no exposure. Despite this significant association, the substantial heterogeneity across studies (I² = 98.08%) suggests that variability in study designs, population characteristics, and methods of cadmium exposure assessment contributed to the differing results, highlighting the need for caution when interpreting these findings. Additionally, while the absence of publication bias strengthens the reliability of the results, the lack of consistent adjustment for key confounders such as smoking, a common source of cadmium exposure, may have influenced the observed association. Future research should focus on improving exposure assessment through biomonitoring, controlling for confounding factors more rigorously, and exploring the biological mechanisms through which cadmium contributes to pancreatic carcinogenesis. Public health efforts should also prioritize reducing cadmium exposure, particularly in high-risk populations, to mitigate its potential impact on pancreatic cancer incidence.

### Conclusion

This systematic review and meta-analysis provide strong evidence that cadmium exposure is associated with an increased risk of pancreatic cancer, with a pooled OR of 2.01 (95% CI: 1.30, 2.72). The subgroup analysis further highlights that the effect of cadmium exposure varies significantly across different study groups, as evidenced by a significant test of group differences (Qb(4) = 30.24, p = 0.00). This suggests that factors such as study design, methods of exposure assessment, and geographic location contribute to the variability in the observed association. Despite substantial heterogeneity (I² = 98.08%), the overall positive association across studies strengthens the conclusion that cadmium is a potential environmental risk factor for pancreatic cancer. Further research is needed to clarify the biological mechanisms underlying this association and to explore strategies for reducing cadmium exposure, especially in high-risk populations.

## Supporting information

S1 File PRISMA checklist. (DOCX)

S2 File Table 1. (PDF)

S3 File Included articles. (RAR)

S4 File All screened references, data extractors and date of data extraction. (CSV)

S5 File All information extracted from included studies. (XLSX)

S6 File Data of analysis (STATA). (XLSX)
