# Supplementary material for: Cadmium exposure and risk of pancreatic cancer: Systematic review and meta-analysis
Source: PLoS One. 2025 Apr 29;20(4):e0319283. doi: 10.1371/journal.pone.0319283 (PMC12040173; doi:10.1371/journal.pone.0319283)
Supplement: S2 File — (PDF) [file pone.0319283.s002.pdf]

Reference	Quality Score	Country	Study Design	Source of funding	Age range	Exposure Assessment Measurement Method	Diagnostic Criteria Used	Follow up Duration	Reported Results(Odds Ratio and95% Confidence Intervals)
Brian G.Luckett (food)**(16)**	Low Risk	USA	case-control	Louisiana State Board of Regents	>20 Years	questionnaire-laboratory examination	laboratory examination(urine sample)	2001 to 2005	OR: 1.90 95% CI: (1.88-2.82)
Brian G.Luckett (urinary cadmium)**(16)**	Low Risk	USA	case-control	Louisiana State Board of Regents	>20 Years	questionnaire-laboratory examination	laboratory examination(urine sample)	2001 to 2005	OR: 5.42 95% CI: (3.02-8.81)
Brian G.Luckett (occupational) **(16)**	Low Risk	USA	case-control	Louisiana State Board of Regents	>20 Years	questionnaire-laboratory examination	laboratory examination(urine sample)	2001 to 2005	OR: 1.65 95% CI: (1.03-1.29)
Brian G.Luckett (smoking)**(16)**	Low Risk	USA	case-control	Louisiana State Board of Regents	>20 Years	questionnaire-laboratory examination	laboratory examination(urine sample)	2001 to 2005	OR: 1.07 95% CI: (0.75-1.42)
Brian G.Luckett (water)**(16)**	Low Risk	USA	case-control	Louisiana State Board of Regents	>20 Years	questionnaire-laboratory examination	laboratory examination(urine sample)	2001 to 2005	OR: 1.51 95% CI: (0.77-2.97)
Brian G.Luckett (living on a farm)**(16)**	Low Risk	USA	case-control	Louisiana State Board of Regents	>20 Years	questionnaire-laboratory examination	laboratory examination(urine sample)	2001 to 2005	OR: 1.56 95% CI: (0.81-3.01)
Andre´ F S Amaral**(17)**	Low Risk	Spain	Case-Control	*	Not mentioned	Interview-questionnaire, clinical history	Hospital	1992-2005	OR:1.59 95% CI: (0.88-3.87)
Vladimir R. Djordjevic**(18)**	Low Risk	Serbia	case-control	The project was partly supported by the Ministry of Education, Science and Technological Development of Serbia and the Oklahoma State University Center for Health Science Pilot Grant Program	All	Laboratory examination	Human observational, experimental and in vitro studies	2014 to 2016	OR: 2.48 95% CI: (0.93-9.22)
Alison M. Kriegel**(19)**	Low Risk	Egypt	case-control	*[2]	All	Laboratory examination and Histopathologic examination, Interview-questionnaire	laboratory examination(serum cadmium)	2001 to 2002	OR: 1.12 95% CI: (1.04-1.23)
Esther García-Esquinas**(20)**	Low Risk	USA	Cohort	*[3]	45-75 years	questionnaire and physical examination	each state's Department of Health	1990 to 2008	OR: 2.00 95% CI: (1.29-3.11)
Scott V Adams**(21)**	Low Risk	USA	Cohort	SVA was supported in part by NIH National Cancer Institute Cancer Prevention Training Grant, and an ASPO/ASCO Cancer Prevention Fellowship sponsored by the Prevent Cancer Foundation	≥17 years	interviews and examinations	Not mentioned	Not mentioned	OR: 1.93 95% CI: (1.50-4.53)
